# CaPDX1, a Novel Protein, Positively Regulates Cold Stress Tolerance via Interaction with CaSnRK2.4 in Pepper (*Capsicum annuum* L.)

**DOI:** 10.3390/ijms27083676

**Published:** 2026-04-20

**Authors:** Altaf Hussain, Qianyi Wang, Yipeng Su, Yuqi Guo, Ikram Ullah, Syed Sohail Ahmad, Nadia Sajjad, Jiangbai Guo, Maira Jahangir, Huafeng Zhang, Rugang Chen

**Affiliations:** College of Horticulture, Northwest A&F University, Yangling 712100, China; altaf@nwafu.edu.cn (A.H.);

**Keywords:** screening, protein–protein interaction, *CaSnRK2.4*, *CaPDX1*, cold tolerance, pepper

## Abstract

*Capsicum annuum* is a Solanaceae crop that is sensitive to cold, which affects its growth and development upon prolonged exposure and ultimately reduces yield. In response, a complex regulatory network of cold-responsive genes is activated. Earlier studies have shown that SnRKs play a positive role in enhancing cold tolerance in different crops, including peppers; however, the underlying molecular mechanisms and downstream targets have yet to be fully elucidated. In this study, yeast hybrid screening using CaSnRK2.4 identified a potential interacting partner CaPDX1. The interaction between CaPDX1 and CaSnRK2.4 was further confirmed through Y2H, luciferase complementation, and bimolecular fluorescence complementation assays. Subcellular localization showed that CaPDX1 and CaSnRK2.4 are localized in the nucleus as well as in the cell membrane. Silencing of *CaPDX1* through VIGS showed increased susceptibility of peppers to cold stress, negatively influenced antioxidant enzymatic activities, and increased relative electrolyte leakage and malondialdehyde levels. Conversely, transient overexpression of *CaPDX1* in peppers enhanced cold tolerance by reducing the accumulation of REL and MDA. Ectopic expression of *CaPDX1* in *Arabidopsis thaliana* significantly improved its cold tolerance, accompanied by enhanced activity of antioxidant enzymes and increased chlorophyll content. In summary, these results indicate that *CaPDX1* is a positive regulator of cold tolerance in pepper, and its mechanism of action involves interaction with *CaSnRK2.4* and the regulation of physiological and molecular responses in pepper under cold stress.

## 1. Introduction

Plant growth, development and productivity are constantly challenged by various environmental factors, including both biotic and abiotic stresses, which seriously affect the normal lifespan of plants and sometimes even lead to plant death [1,2]. Pepper (*Capsicum annuum* L.), a highly important economical vegetable crop worldwide, is very sensitive to chilling temperatures, which significantly reduce its growth, development, and productivity [3,4,5]. Cold stress disrupts cellular homeostasis by damaging membrane integrity, impairing enzyme activities, and triggering the excessive accumulation of reactive oxygen species, leading to physiological and biochemical disturbances that ultimately cause tissue injury and stunted growth [6,7]. To cope with such adverse environmental conditions, plants have evolved intricate stress response mechanisms, including signal sensing, transcriptional regulation, and downstream protective responses [4,8]. Adaptation to stress depends on interconnected signaling pathways involving various regulatory and structural proteins among which transcription factors (TFs) play a central role by modulating the expression of stress-responsive genes through binding to specific cis-acting elements in target promoters [9,10,11]. Understanding and addressing the molecular mechanisms of tolerance to abiotic stresses requires the identification and functional characterization of stress-related partner genes [12]. Cold stress has been shown to induce the expression of multiple TF families, including C-repeat binding factors (CBFs), APETALA2 (AP2), basic leucine zipper (bZIP), NAC, MYB proteins and basic helix–loop–helix (bHLH), which collectively orchestrate the transcriptional regulation of genes involved in stress protection and adaptation [13,14]. SnRK2s are a widely explored protein kinase family that plays crucial roles in plant responses to different abiotic stresses, particularly through ABA-dependent signaling pathways. For instance, Arabidopsis, *OST1/SnRK2.6* positively regulates freezing tolerance by phosphorylating and stabilizing ICE1, thereby activating downstream CBF-dependent cold-responsive genes [15]. In our previous study in pepper, *CaSnRK2.4*, a member of the ABA-responsive SnRK2 kinase family, was shown to positively regulate cold tolerance by modulating phosphorylation signaling, ABA accumulation, and ROS homeostasis [4]. Silencing of *CaSnRK2.4* significantly increased chilling sensitivity, highlighting its important role in pepper cold stress signaling. Given the positive role of SnRK-mediated signaling in pepper cold tolerance, we speculated that its downstream target genes may also contribute to cold stress adaptation. To identify such candidates, downstream screening was performed, leading to the discovery of CaPDX1 as a potential gene. Previous studies in Arabidopsis thaliana have found that *AtPDX1* is crucial for the biosynthesis of vitamin B6 and is involved in its healthy growth, development and especially provides tolerance in response to abiotic stresses, suggesting that PDX family proteins play a broader role in stress responses [16,17]. As another example, the expression of PDX1 and PDX2 orthologs in Arabidopsis is upregulated under cold stress and other abiotic stressors, underscoring the broader involvement of PDX family members in plant stress adaptation [18]. Beyond cold stress, multiple studies have reported that fluctuations in vitamin B6 levels and associated gene expression occur under diverse stress conditions, implying that PDX-mediated PLP metabolism participates in oxidative stress defense [19]. Another study of genome-wide analyses in Brassica napus, further shows conservation and stress responsiveness of the PDX gene family, underlining the evolutionary importance of PDX members in stress adaptation [20]. Despite these advances in model and crop plants, the protein–protein interaction networks and downstream regulatory mechanisms underlying cold stress responses in pepper remain poorly understood. This study aims to explore cold stress regulatory mechanisms in pepper by screening and validating an interacting partner of a sucrose non-fermenting 1-related protein kinase (CaSnRK2.4). Through yeast two-hybrid screening, CaPDX1 was identified as a putative interacting partner of CaSnRK2.4. This interaction was subsequently validated using luciferase complementation imaging, bimolecular fluorescence complementation, and complementary yeast-based assays. Functional analyses revealed that *CaPDX1* acts as a positive regulator of cold stress tolerance. Virus-induced gene silencing of *CaPDX1* significantly reduced cold tolerance in pepper, while transient overexpression in pepper and stable transformation in *A. thaliana* enhanced cold stress resistance. Collectively, these results reveal a previously uncharacterized role for *CaPDX1* in cold stress regulation and provide new insights into the molecular networks governing cold tolerance in pepper.

## 2. Results

### 2.1. Characterization of CaPDX1 and Bioinformatic Analysis

**Figure 1:**
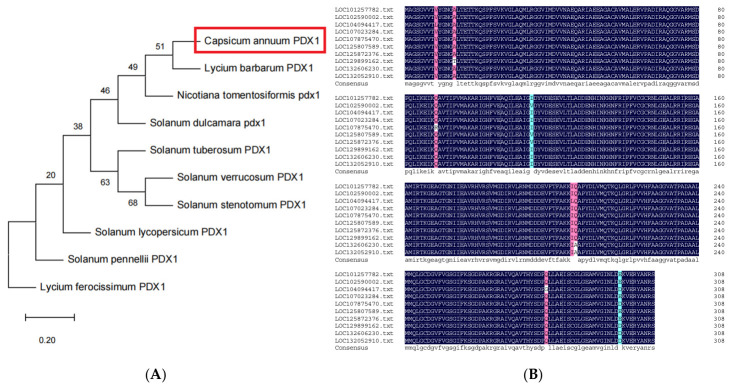
(**A**) Phylogenetic analysis of CaPDX1 with PDX proteins from various plant species. (**B**) Multiple sequence alignment of CaPDX1 and other plant PDX members, showing conserved regions in black with 100% identity, pink with ≥75% identity, and blue with ≥50% identity.

For bioinformatic analysis, the *CaPDX1* gene (LOC107875470) was used as the query. A phylogenetic tree was constructed using DNAMAN (version 7) software to assess the evolutionary relationships between CaPDX1 and PDX proteins from various plant species [21]. The analysis indicated that CaPDX1 is highly conserved within the Solanaceae family, showing closest homology to other Solanaceae PDX proteins (Figure 1A). To further characterize the structural functions of CaPDX1, multiple sequence alignment was performed with PDX from diverse species using DNAMAN (Figure 1B). The alignment showed highly conserved motifs across different species, highlighting the evolutionary conservation of CaPDX1 and its potential functional significance.

### 2.2. CaPDX1 Physically Interacts with CaSnRK2.4

**Figure 2:**
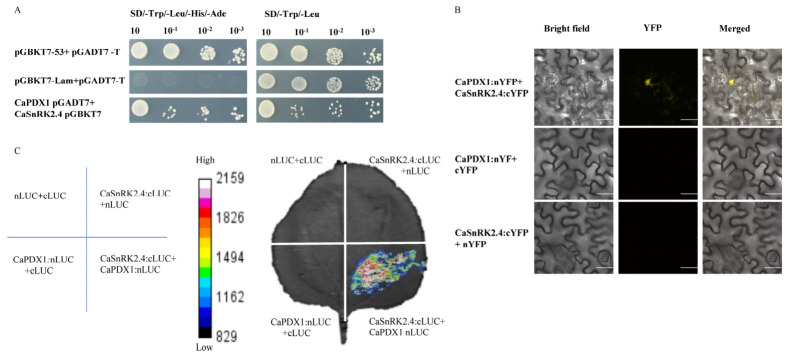
Physical interaction between CaSnRK2.4 and CaPDX1. (**A**) Yeast two-hybrid (Y2H) assays confirm the interaction between CaSnRK2.4 and CaPDX1, and pGADT7-AD + pGBKT7-Lam and pGADT7-AD + pGBKT7-53 served as negative and positive controls, respectively. (**B**) BiFC analysis demonstrates the interaction of CaSnRK2.4 and CaPDX1 in the nucleus of plant cells. Scale bar = 30 μm. (**C**) Luciferase complementation imaging (LCI) assay shows the interaction between CaSnKR2.4 and CaPDX1 in *N. benthamiana* leaves.

To investigate the molecular mechanism underlying CaPDX1 function under cold stress, yeast two-hybrid (Y2H) screening was performed, which identified CaSnRK2.4 as a potential interacting partner of CaPDX1 (Figure S1). It was further confirmed by Y2H assays, as yeast co-expressing both proteins grew on selective media, whereas controls showed no growth, indicating a specific interaction (Figure 2A). In addition, bimolecular fluorescence complementation (BiFC) assays in *N. benthamiana* leaf cells were performed to validate the interaction. Co-expression of CaPDX1-nYFP with CaSnRK2.4-cYFP produced strong YFP fluorescence, primarily interacts in the Nucleus and as well as light signal in cytoplasm, confirming the physical interaction of these proteins. No fluorescence was observed in negative controls expressing either protein with empty vectors, indicating the specificity of the interaction (Figure 2B). Furthermore, a luciferase complementation assay corroborated these findings. Co-expression of CaPDX1-nLUC and CaSnRK2.4-cLUC in *N. benthamiana* leaves generated a strong luminescent signal, whereas combinations with empty vectors showed minimal background luminescence (Figure 2C). Collectively, these results demonstrate that CaPDX1 physically interacts with CaSnRK2.4.

### 2.3. Subcellular Localization of CaPDX1 and CaSnRK2.4

**Figure 3:**
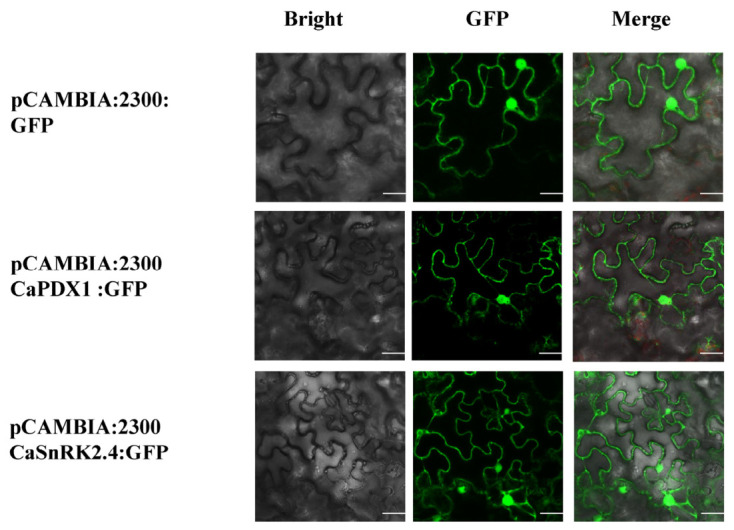
Subcellular localization of CaPDX1 and CaSnRK2.4 in *N. benthamiana* leaves. Scale bar = 30 μm.

For subcellular localization analysis, the in-frame coding sequence was fused to the green fluorescent protein (GFP) reporter under the full control of the 35S promoter, generating pCAMBIA:2300+CaPDX1:GFP and pCAMBIA:2300+CaSnRK2.4:GFP constructs. Both (pCAMBIA:2300+CaPDX1:GFP, pCAMBIA:2300+CaSnRK2.4:GFP) and control (pCAMBIA:2300::GF) were transiently expressed in the leaves of *N. benthamiana*. Results under confocal microscopy confirmed both the proteins, CaPDX1 and CaSnRK2.4 are located in the nucleus and as well as in the cell membrane, while the control distributed throughout the cell (Figure 3).

### 2.4. Silencing of CaPDX1 Decreases Tolerance to Cold Stress

**Figure 4:**
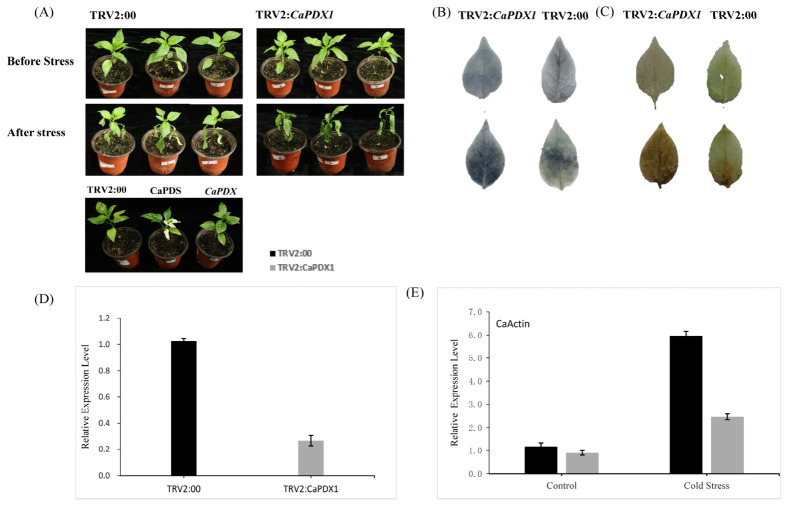
Silencing of *CaPDX1* compromises cold stress tolerance in pepper. (**A**) Phenotypic response of TRV2:*CaPDX1* and control plants after exposure to 4 °C for three days. TRV2:CaPDX1 plants exhibit more-severe cold-induced damage. (**B**) NBT staining indicating superoxide (O_2_^−^) accumulation. (**C**) DAB staining showing hydrogen peroxide (H_2_O_2_) accumulation. (**D**) Relative expression of *CaPDX1* in VIGS and control plants. (**E**) Expression of the reference gene CaActin across treatments.

To investigate the role of CaPDX1 in cold stress tolerance in pepper, we generated virus-induced gene silencing (VIGS) lines in the P70 cultivar. A 300 bp CDS fragment of *CaPDX1* was cloned into the TRV2 vector to construct TRV2:*CaPDX1*. Then constructs TRV2:*CaPDX1*, TRV2:00 and TRV2:*CaPDS* were transformed into *A. tumefaciens* GV3101. Two-week-old pepper seedlings were infiltrated with TRV2:*CaPDX1*, TRV2:00 (negative control), and TRV2:*CaPDS* (positive control). Approximately 4 weeks post-infiltration, TRV2:*CaPDS* plants exhibited characteristic leaf bleaching, whereas TRV2:00 plants showed no visible phenotype. Notably, TRV2:*CaPDX1* plants exhibited moderate leaf bleaching, indicating effective gene silencing (Figure 4A). To assess cold stress tolerance, VIGS and control seedlings were exposed to 4 °C for three days. TRV2:*CaPDX1* plants displayed more-severe damage than the control plants (Figure 4A), suggesting that silencing of CaPDX1 compromises cold tolerance. Histochemical staining using DAB and NBT was performed to estimate H_2_O_2_ and O_2_^−^ accumulation, respectively. After cold treatment, both DAB and NBT staining were more pronounced in TRV2:*CaPDX1* plants than in controls, with larger staining areas and darker coloration, indicating elevated reactive oxygen species (ROS) accumulation in silenced plants (Figure 4B,C). These findings suggest that this novel gene, *CaPDX1*, has a positive effect on the cold stress tolerance of chili peppers, possibly by regulating ROS homeostasis. Gene silencing was confirmed through qRT-PCR performed after 2 weeks of infiltration. The results and analysis in TRV2:*CaPDX1* plants confirmed the expression level was reduced by approximately 78%, with a relative expression level of approximately 0.2 compared with the control plants (Figure 4D). The relative expression level of the reference gene *CaActin* was determined before and after stress, which confirmed the *CaPDX1* silencing expression in cold stress (Figure 4E).

**Figure 5:**
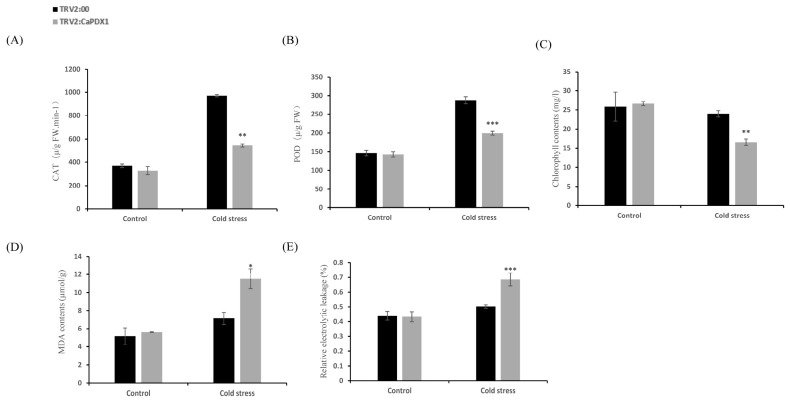
Analysis of physiological and biochemical responses in pepper plants under cold stress. (**A**) CAT activity. (**B**) POD activity. (**C**) Chlorophyll content. (**D**) MDA content. (**E**) Relative electrolyte leakage (REL). *p* value is represented as (* *p* < 0.05, ** *p* < 0.01 and *** *p* < 0.001).

Assessing the further role of *CaPDX1* in response to cold stress in pepper plants, we measured the key activities of the ROS-scavenging enzymes, catalase (CAT) and peroxidase (POD), in control and *CaPDX1*-silenced plants under normal and cold stress conditions. Under normal conditions, no significant differences were recorded in enzyme activities between the two groups. Upon exposure to cold stress, CAT and POD activities increased in both control and *CaPDX1*-silenced plants; however, this induction was recorded to be higher in control (TRV2:00) plants than in the silenced lines (Figure 5A,B). Chlorophyll content analysis disclosed no significant difference between control and *CaPDX1*-silenced plants under the normal situation. After cold stress, control plants maintained higher chlorophyll levels than the silenced plants (Figure 5C). Consistently, an indicator of malondialdehyde (MDA) content was significantly elevated in CaPDX1-silenced plants relative to controls (Figure 5D). Similarly, relative electrolytic leakage (REL) showed no difference under normal conditions, but, following cold stress, *CaPDX1*-silenced plants exhibited greater membrane damage than controls (Figure 5E).

### 2.5. Overexpression of CaPDX1 in Arabidopsis Enhances Tolerance to Cold Stress

**Figure 6:**
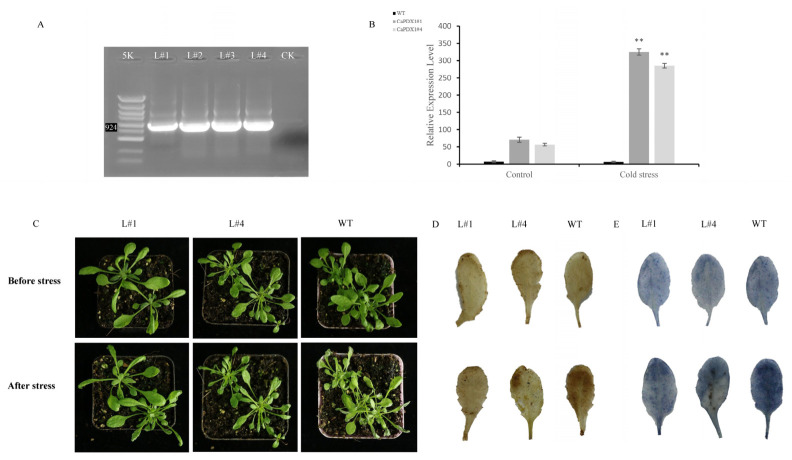
Overexpression of *CaPDX1* enhances cold stress tolerance in Arabidopsis. (**A**) Phenotypic response of *CaPDX1*-overexpressing and wild-type Arabidopsis plants under cold. (**B**) Nitro blue tetrazolium (NBT) staining. ** *p* < 0.01. (**C**) Diaminobenzidine (DAB) staining. (**D**) PCR confirmation of CaPDX1 overexpression in transgenic lines. (**E**) Relative transcript levels of CaPDX1-overexpressed and WT lines.

To investigate the role of *CaPDX1* in response to cold stress, the complete CDS of *CaPDX1* was cloned into the binary vector pCAMBIA:2300 under the control of the CaMV35S promoter. T0 seeds were screened on Kanamycin resistance medium and then lavish green seedlings were transferred into the soil (Figure S2), T1 plants were selected for DNA extraction from healthy lines for further confirmation of successful transformation via PCR. PCR analysis identified L1, L2, L3, and L4 as *CaPDX1*-overexpressing lines (Figure 6A). Two lines (L#1 and L#4) were selected based on their high expression level, superior phenotypical appearance and subsequently used for further experiments. Afterwards we exposed four-week-old T2 transgenic plants and wild-type (WT) controls to cold stress at 4 °C for 3 days. WT plants exhibited pronounced wilting, whereas *CaPDX1*-overexpressing lines (L#1 and L#4) maintained healthier growth, indicating enhanced cold tolerance (Figure 6C). To further elucidate the role of *CaPDX1* in regulating cold tolerance, we assessed physiological responses in leaves of WT and *CaPDX1*-overexpressing transgenic plants before and after cold stress. Reactive oxygen species (ROS) accumulation was evaluated using DAB and NBT staining. No significant differences in DAB or NBT staining under control conditions were observed between WT and transgenic plants. In contrast, following cold stress treatment, transgenic plants exhibited markedly reduced DAB and NBT staining compared with WT plants, indicating lower ROS accumulation in *CaPDX1* OE lines (Figure 6D,E). These results suggest that CaPDX1 enhances cold tolerance by mitigating oxidative stress in Arabidopsis. Relative expression levels confirmed higher expression in *CaPDX1*-overexpressing lines than in WT under cold conditions (Figure 6B). Additionally, seedling growth assays were performed on MS½ kanamycin; no differences were observed on MS 0 kanamycin medium in WT and *CaPDX1*-overexpressed seed germination, while *CaPDX1*-overexpressing lines (1# and 4#) displayed significantly better growth than WT on MS½ kanamycin medium (Figure S3).

**Figure 7:**
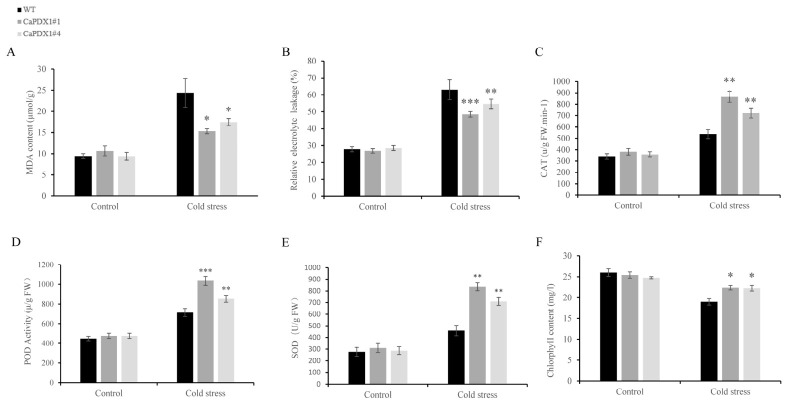
Physiological and biochemical responses of *CaPDX1* in overexpression. (**A**) (MDA) Malondialdehyde content. (**B**) Relative electrolytic leakage. (**C**) CAT activity. (**D**) Peroxidase (POD) activity. (**E**) SOD activity. (**F**) Chlorophyll content. *p* value is represented as (* *p* < 0.05, ** *p* < 0.01 and *** *p* < 0.001).

Furthermore, we assessed lipid peroxidation and membrane stability by measuring malondialdehyde (MDA) contents and relative electrolyte leakage (REL). Both parameters increased under cold stress in WT and transgenic plants (Figure 7A,B); however, the increases were markedly lower in *CaPDX1*-overexpressed plants than in WT. Antioxidant enzyme activities of catalase (CAT), peroxidase (POD), and superoxide dismutase (SOD) were comparable under normal conditions between transgenic and WT plants. Upon exposure to cold stress, *CaPDX1* transgenic lines showed a significant enhancement in CAT, POD, and SOD activities relative to WT (Figure 7C–E). Under control conditions, chlorophyll content experiments showed no significant difference between *CaPDX1*-overexpressing lines (L#1 and L#4) and wild type. But, in comparison, the wild-type plants showed a decrease in chlorophyll content after cold treatment, while transgenic lines maintained significantly higher levels (Figure 7F). In conclusion, these findings clarified that *CaPDX1* overexpression enhances the cold resistance of Arabidopsis thaliana, possibly by improving antioxidant defense capabilities and maintaining chlorophyll content.

### 2.6. Transient Expression of CaPDX1 Enhanced Pepper Cold Tolerance

**Figure 8:**
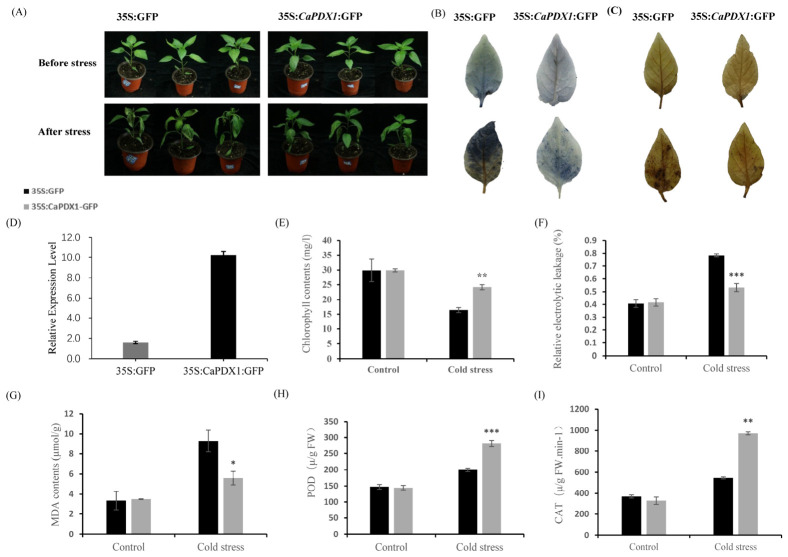
Transient overexpression of *CaPDX1* enhances resistance in pepper plants to cold stress. (**A**) Phenotypic comparison of CaPDX1-overexpressed plants with control plants under cold stress. (**B**) NBT Activity. (**C**) Diaminobenzidine (DAB) activity. (**D**) Relative expression level. (**E**) Chlorophyll content. (**F**) Relative electrolytic leakage. (**G**) MDA content. (**H**) POD activity. (**I**) CAT activity. *p* value is represented as (* *p* < 0.05, ** *p* < 0.01 and *** *p* < 0.001).

To investigate the function of *CaPDX1* in cold stress tolerance, transient overexpression of 35S::*CaPDX1* was performed in pepper leaves.. There were no phenotypic differences observed in normal growth conditions between the 35S::GFP control plants and *CaPDX1*-transient-overexpression (*CaPDX1*-TO) plants. But, after exposure to 48 h of cold stress, leaves of control plants exhibited severe damage, whereas *CaPDX1*-TO leaves showed markedly improved tolerance (Figure 8A). DAB and NBT staining revealed no significant differences between *CaPDX1*-TO and control plants under normal conditions. Upon cold stress, however, the stained areas in *CaPDX1*-TO plants were significantly smaller than those in control plants, indicating reduced accumulation of reactive oxygen species (ROS) (Figure 8B,C). Consistently, RT-qPCR analysis confirmed a pronounced upregulation of *CaPDX1* expression in *CaPDX1*-TO plants compared with controls (Figure 8D). Furthermore, after cold stress, the chlorophyll content of *CaPDX1*-TO plants was higher than that of the control plants (Figure 8E). The relative electrolyte leakage rate (REL, an indicator of membrane damage) was significantly lower in *CaPDX1*-TO plants than in the control plants (Figure 8F). Similarly, malondialdehyde (MDA) levels, an indicator of lipid peroxidation, showed no difference under normal conditions but were significantly reduced in *CaPDX1*-TO plants under cold stress (Figure 8G). Antioxidant enzymes activities of peroxidase (POD) and catalase (CAT) were comparable between transient overexpressed and control pepper plants under normal conditions. Upon exposure to cold stress, *CaPDX1*-transient-overexpressed pepper plants exhibited a significant enhancement in POD and CAT activities relative to control plants (Figure 8H,I). Collectively, these results demonstrate that transient overexpression of *CaPDX1* enhances cold stress tolerance in pepper, likely through mitigating ROS accumulation and preserving membrane integrity.

## 3. Discussion

Plants have evolved sophisticated and unique regulatory networks to survive and defend against unwanted environmental conditions, including cold stress, which extremely reduce plant growth, development, and crop productivity [22,23,24]. Cold stress not only disrupts cellular homeostasis, impairs photosynthesis, and induces oxidative damage but ultimately effect plant healthy growth, productivity, and high yield [7,25,26]. Earlier studies and research have discovered that numerous protein kinases (SnRKs) play a great role in plant stress signaling and overall protection [27,28,29]. In pepper, CaSnRK2.4 has been reported to function as a positive regulator of cold stress responses [4]. However, the downstream regulatory components associated with CaSnRK2.4-mediated cold tolerance remained unclear.

In the present findings, CaPDX1 was identified as an interactive protein of CaSnRK2.4 through yeast two-hybrid screening and was further validated using well-established unique methods such as Y2H, luciferase complementation, and BiFC assays. Subcellular localization analyses revealed that CaSnRK2.4 is localized in the nucleus as well as in the cell membrane, consistent with its role in signal perception and transcriptional regulation. In contrast, CaPDX1 is also distributed in both the nucleus and cell membrane, suggesting that CaPDX1 may be involved in signal transduction between extracellular stress perception and nuclear gene regulation. This spatial distribution supports the view that CaPDX1 is a downstream component of the CaSnRK2.4-mediated cold signaling pathway.

Functional analysis using virus-induced gene silencing (VIGS) in pepper and overexpression in pepper and Arabidopsis confirmed that *CaPDX1* plays a positive role in cold tolerance. Silencing of *CaPDX1* in pepper significantly increased its sensitivity to low-temperature stress, while transient overexpression in pepper and stable expression in Arabidopsis enhanced its cold tolerance. Under low-temperature stress, *CaPDX1*-silenced plants exhibited decreased photosynthetic capacity, increased membrane damage, and enhanced oxidative stress, indicating that *CaPDX1* is essential for maintaining physiological stability under low-temperature stress.

Photosynthesis is highly sensitive to cold stress, which severely affects plant growth; reduced photosynthetic efficiency is a common consequence of chilling conditions [30,31]. In this study, *CaPDX1*-overexpressing plants maintained higher chlorophyll content and improved photosynthetic performance under cold stress, whereas *CaPDX1*-silenced plants exhibited a pronounced inhibition of photosynthetic activity. These findings suggest that *CaPDX1* contributes to cold tolerance by protecting the photosynthetic machinery.

Oxidative stress also severely affects plant growth, contributing to cold-induced cellular damage and injuries [32,33]. This stress leads to excessive accumulation of reactive oxygen species (ROS), resulting in lipid peroxidation and membrane injury [34,35]. Malondialdehyde (MDA) and electrolyte leakage (REL) are commonly used indicators of membrane damage in plants. Therefore, CaPDX1-silenced plants accumulated higher levels of MDA and exhibited increased REL, whereas CaPDX1-overexpressing plants showed significantly reduced MDA content and REL, indicating improved membrane integrity under cold stress.

Consistent with these observations, antioxidant enzyme activities were markedly affected by *CaPDX1* expression. Activities of (SOD) superoxide dismutase, (POD) peroxidase, and catalase (CAT) were highly reduced in CaPDX1-silenced plants, resulting in excessive ROS accumulation, as confirmed by DAB and NBT staining. In contrast, *CaPDX1*-overexpressing pepper and Arabidopsis plants exhibited enhanced antioxidant enzyme activities and reduced ROS levels, indicating a more efficient ROS scavenging system. These findings suggest and confirm that *CaPDX1* enhances cold tolerance, at least in part, by strengthening antioxidant defense and maintaining cellular redox homeostasis.

Multiple research and findings have demonstrated that PDX family genes are involved in plant responses to abiotic stresses, including cold, drought, and salinity, and are often regulated by hormone signaling pathways such as abscisic acid (ABA) [36,37]. In this study, *CaPDX1* expression was strongly induced by cold stress suggesting that *CaPDX1* may function at the intersection of stress. The enhanced cold tolerance observed in *CaPDX1* OE plants and the increased sensitivity of *CaPDX1*-silenced plants further support this hypothesis. In summary, our findings demonstrate that *CaPDX1* functions as a positive regulator of cold stress tolerance in pepper. Through its interaction with CaSnRK2.4, CaPDX1 enhances cold resistance by maintaining photosynthetic capacity, preserving membrane integrity, and activating antioxidant defense systems. This study provides new insights into the CaSnRK2.4–CaPDX1 regulatory module in cold stress responses and highlights *CaPDX1* as a promising genetic target for improving cold tolerance in pepper and other plants.

## 4. Materials and Methods

### 4.1. Plant Material and Growing Conditions

All the materials were obtained from Northwest A&F University, Yangling, Shaanxi, China including pepper seeds of cultivar “P70”, Arabidopsis and Tobacco (*N. benthamiana*). Seeds were sown in a nutrient-rich soil medium and grown under controlled conditions. Pepper plants were maintained at 25 °C conditions with a 16 h light and 8 h dark photoperiod [8,38]. Tobacco plants were sown in soil and then kept in a growth chamber at 22 °C/18 °C, (day/night) under a 16 h light and 8 h dark cycle and 60% relative humidity [39]. The Arabidopsis seeds were sterilized through 20% NaClO and 70% ethanol and then germinated on Murashige and Skoog (MS) medium (PhytoTechnology Laboratories, Shawnee Mission, KS, USA). They were then exposed to 4 °C for 2 days and shifted to a growth chamber maintained at 22/18 °C (day/night) under a 16/8 h light and dark cycle for healthy growth and development.

### 4.2. RNA Extraction and qRT-PCR Analysis

Total RNA was extracted using the RNA extraction kit from Tiangen Biotech Co., Ltd. (Beijing, China), and cDNA was synthesized using the cDNA synthesis kit (Takara Bio Inc., Beijing, China) according to the manufacturer’s instructions and the method followed was previously described in our earlier studies [40]. qRT-PCR was carried out using the method of SYBR Premix Ex Taq II (TaKaRa, Dalian, China). The 2^−ΔΔCT^ method was carried out for relative gene expression levels, with *CaActin* serving as an internal control gene [41].

### 4.3. Sequences Retrieval, Cloning, Bioinformatic Analysis of CaPDX1

A blast search was performed on 24 January 2024, to retrieve the full-length cDNA sequence of *CaPDX1* (CA06g25350) from the pepper genome database (https://solgenomics.net/tools/blast/) using the corresponding protein sequence. For amplification the gene-specific primers were designed with forward primer ATGGCCGGAAGTGGCGTTGTAACAG and reverse primer CACTCAGAACGATTAGCATACCTC. The amplified PCR fragments were ligated into the pMD19-T cloning vector (TaKaRa, China) and subsequently verified by DNA sequencing (Shanghai GeneCore Biotechnologies Co., Shanghai, China). For phylogenetic and multisequence analysis, the top 10 homologs of CaPDX1 with 100% query coverage and ≥80% sequence similarity were downloaded from the NCBI database (https://www.ncbi.nlm.nih.gov/genbank/, accessed on 5 March 2026).

### 4.4. Subcellular Localization of CaPDX1 and CaSnRK2.4

The CDS sequence (Table S1) without the stop codon was cloned into the pCAMBIA:2300::GFP vector to generate the pCAMBIA:2300:CaPDX1::GFP and pCAMBIA:2300:CaSnRK2.4::GFP constructs, and the empty vector pCAMBIA:2300::GFP was taken as a control for subsequent experiments. These constructs were then transformed by following the lab standard protocol into the Agrobacterium tumefaciens strain GV3101 and at a subsequent time infiltrated into *N. benthamiana* leaves. The infiltrated plants were maintained at 22/18 °C (day/night) for 2 days, after which GFP fluorescence was observed through a fluorescence microscope (Olympus, Tokyo, Japan) following the method described by [40,42].

### 4.5. Transient Gene Expression Assays in Pepper

The CDS of *CaPDX1* (Table S1), without stop codon, was amplified and cloned into the 35S::GFP plasmid vector for transient overexpression. Confirmation of recombinant plasmids was carried out through sequencing and then transformed into A. tumefaciens strain GV3101 by following the lab standard protocol [43]. The 28-day-old pepper plants were infiltrated with the transformed Agrobacterium suspension and maintained in the dark for 12 h, after which they were kept at normal growth conditions. Seventy-two hours post-infiltration, plants were exposed to 4 °C cold stress for 48 h. Transient expression and relative expression of CaPDX1 in infiltrated plants was verified by qPCR. Cold-related indexes were recorded after three days of infiltration.

### 4.6. Stable Transformation and Generation of Arabidopsis OE Lines

The CDS sequence was amplified from the P70 pepper line using gene-specific forward and reverse primers. The amplified fragment was cloned into the overexpression vector pCAMBIA:2300:*CaPDX1* under the CaMV35S promoter and subsequently transformed into A. tumefaciens strain GV3101 [44]. The ectopic transformation was carried out using the established floral dip method, followed by multiple trials. Transgenic seeds were screened and confirmed based on resistance to kanamycin, and then T2-generation plants were used for further physiological and biochemical analysis.

### 4.7. Virus-Induced Gene Silencing (VIGS)

A 300 bp fragment of its ORF was cloned into the tobacco rattle virus-based 2 (TRV2) vector. Then, recombinant vector (TRV2-*CaPDX1*), along with TRV2:*CaPDS* (positive control), and TRV2:00 (negative control) were transformed into A. tumefaciens strain GV3101. Two-week-old pepper seedlings were infiltrated at the cotyledon stage with Agrobacterium strains carrying TRV2:*CaPDX1*, TRV2:*CaPDS* (positive control), or TRV2:00 (negative control). After infiltration, plants were kept in the dark for 48 h and then placed in normal growth conditions according to the laboratory protocol [27]. Gene silencing efficiency was assessed at 28 days post-infiltration using qPCR. After gene silencing confirmation other cold related experiment was performed.

### 4.8. Biochemical Analyses, Histochemical Staining and Cold Stress Assays

The relative electrolyte leakage (REL) analysis was measured according to the previously established protocols [45]. Malondialdehyde (MDA) content was determined using the thiobarbituric acid (TBA) method as reported earlier [46]. Briefly, 0.5 g of leaf tissue was ground in 1 mL of trichloroacetic acid (TCA) 10% and diluted to 10 mL with TCA. The homogenate was centrifuged at 12,000× *g* for total of 10 min at 4 °C, and the 0.6% TBA solution was mixed with supernatant and boiled for 15 min. Data was measured at 600, 532, and 450 nm to calculate the MDA content. The total chlorophyll content was measured following the procedure of Arkus et al. [47]. Histochemical staining of O_2_^−^ and H_2_O_2_ was executed with DAB and NBT, respectively, following the protocol of Kim et al. [48]. For cold stress tolerance assays, CaPDX1-silenced, overexpressing, and control pepper and Arabidopsis plants were exposed to 4 °C for three days, and physiological responses were subsequently assessed.

### 4.9. Y2H Library Screening of PDX1 from CaSnRK2.4

The full-length CDS of CaSnRK2.4 (Table S1) was amplified and cloned into the prospective vector pGBKT7 to form the bait construct. The bait plasmid (CaSnRK2.4-pGBKT7) was transformed into the Y2HGold strain along with a pepper cDNA library cloned into the pGADT7 vector. Co-transformed yeast cells were cultured on selective SD-Trp-Leu-Ade-His (-T-L-A-H) media plates, following the protocol of Zhang et al. [39,49]. These plates were then incubated at 30 °C for 3–5 days. Colonies showing robust growth were screened by PCR using T7 and 3′AD primers, and the amplified products were sequenced commercially for the identification of putative interacting partners.

### 4.10. Yeast Two-Hybrid (Y2H) Assay

The interaction between CaPDX1 and CaSnRK2.4 was verified by using the full-length CDS of CaPDX1 (Table S1) and cloned into the pGADT7 vector to generate the AD construct, while CaSnRK2.4 was cloned into pGBKT7 to generate the BD construct, following standard methods with minor modifications [43,50]. The Y2H assay was performed according to the ShaanXi Pyeast Bio. Co., Ltd., Xi’an, China, protocol. Briefly, yeast strains were transformed with the recombinant constructs, ensuring the bait plasmid was non-toxic and lacked autoactivation. Co-transformed yeast cells were initially cultures on SD/-Leu/-Trp medium plates to select for successful growth. Positive interactions were then assessed by growth on SD/-Ade/-His/-Leu/-Trp medium, indicating physical interaction between the bait and prey proteins.

### 4.11. Bimolecular Fluorescence Complementation (BiFC) Assay

For BiFC assays, the coding sequence of CaPDX1 was inserted into the special vector 35S-SPYNE for the construction of CaPDX1-nYFP, while the CDS of CaSnRK2.4 was inserted into the 35S-SPYCE vector to construct CaSnRK2.4-cYFP. The constructs were then introduced into the GV3101 *A. tumefaciens* strain following the established methods described previously [39,49]. *N. benthamiana* leaves (4 weeks old) were infiltrated with the following combinations: CaPDX1-nYFP + CaSnRK2.4-cYFP for interaction assays, CaPDX1-nYFP + cYFP:00 and CaSnRK2.4-cYFP + nYFP:00 as negative controls. After infiltration, plants were maintained in the dark for 12 h, followed by 48 h under normal growth conditions. Florescence was observed by using a confocal laser scanning microscope (TCS-SP8; Leica, Wetzlar, Germany).

### 4.12. Luciferase Complementary Imaging Assay

To confirm the interaction via luciferase complementation assay, the full-length CDS sequence of CaSnRK2.4 was inserted into the vector, pCAMBIA-cLUC, and the CDS sequence of CaPDX1 was inserted into the vector pCAMBIA-nLUC, with nLUC and cLUC serving as controls. The empty and recombinant plasmids were introduced into *A. tumefaciens* GV3101 cell. Subsequently, 4-week-old leaves of tobacco, *N. benthamiana*, were infiltrated with the following combinations: pCAMBIA-nLUC:CaPDX1+pCAMBIA-cLUC:CaSnRK2.4 for interaction assays, and pCAMBIA-cLUC:CaSnRK2.4+ nLUC and pCAMBIA-nLUC:CaPDX1 + cLUC as negative controls. After infiltration, plants were incubated for 12 h in the dark, followed by 48 h under normal growth conditions. CCD imaging system (Night OWL II LB983, Berthold Technologies, Bad Wildbad, Germany) luminescence signals were used for capture and quantification.

### 4.13. Statistical Analysis

All data are presented as the mean ± standard error (SE) of three independent biological replicates. Significant differences compared with the control group were determined by one-way ANOVA at *p* ≤ 0.05.

## 5. Conclusions

Screening and validation of CaPDX1 revealed its crucial role in chili peppers for regulating their cold tolerance and confirmed it as a downstream component of the CaSnRK2.4-mediated cold signaling pathway. Functional analysis using virus-induced gene silencing and overexpression further confirmed that this gene not only protects chili peppers from severe damage under low-temperature conditions but also enhances their cold tolerance by maintaining plasma membrane integrity, reducing cell damage, and promoting antioxidant enzyme activity. This discovery provides breeders with a new perspective on understanding the molecular network of chili pepper cold tolerance. Further study is required to confirm and elucidate the detailed regulatory mechanisms of CaPDX1, particularly its role in responding to abiotic stresses in other crops. In brief, this study provides a promising genetic target for improving the cold tolerance of chili peppers and other crops through molecular breeding and genetic engineering.

## Data Availability

The data including sequence information and other supporting information are included in this main article and Supplementary Materials. Further inquiries can be directed to the corresponding authors.

## Supplementary Materials

The following supporting information can be downloaded at https://www.mdpi.com/article/10.3390/ijms27083676/s1.
