# Supplementary material for: CaPDX1, a Novel Protein, Positively Regulates Cold Stress Tolerance via Interaction with CaSnRK2.4 in Pepper (Capsicum annuum L.)
Source: Int J Mol Sci. 2026 Apr 20;27(8):3676. doi: 10.3390/ijms27083676 (PMC13116668; doi:10.3390/ijms27083676)
Supplement: Supplementary file 1 [file ijms-27-03676-s001.zip › ijms-4232839-supplementary.pdf]

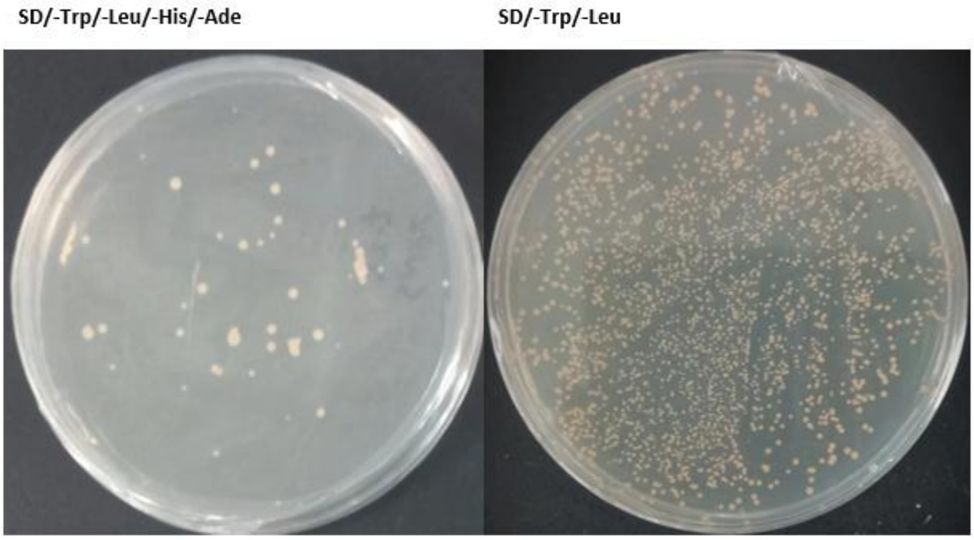

Figure S1: Screening of CaPDX1 from *CaSnRk2.4* through Y2H Screening.

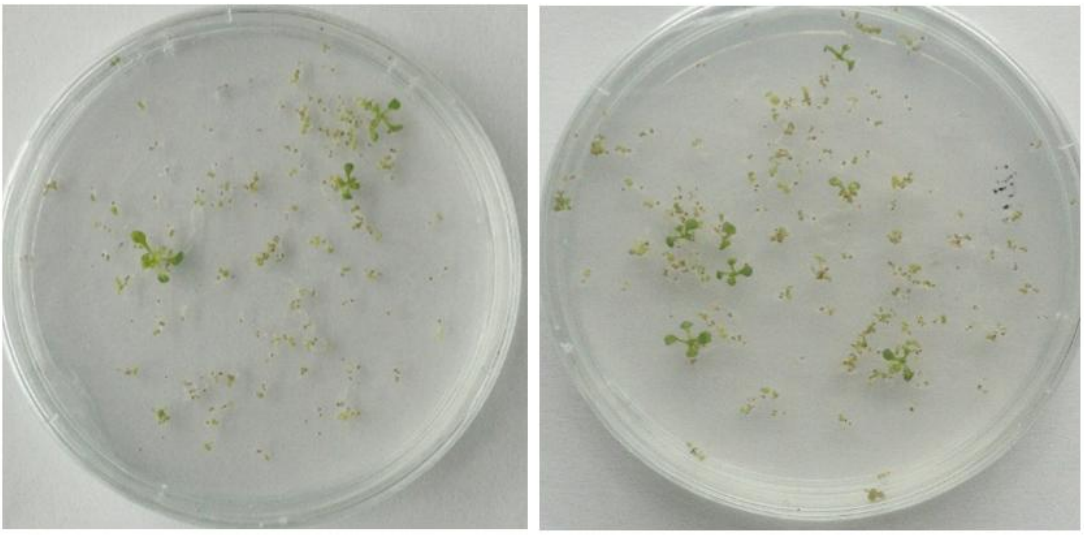

Figure S2: Screening of Arabidopsis seeds on MS kanamycin ½ medium.

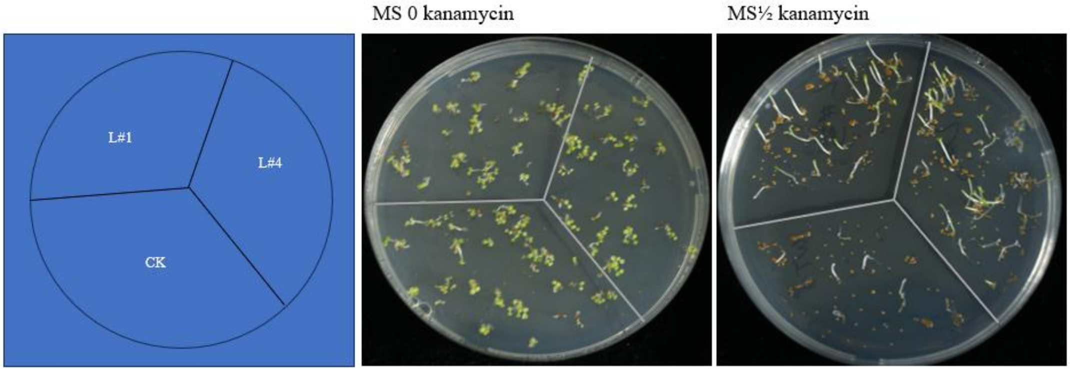

Figure S3: Germination of Arabidopsis, *CaPDX1* overexpressed and WT lines seeds on MS kanamycin ½ and simple MS medium.

S.No	Name		Forward	Reverse
1.	CaPDX1	AD	ATGGCCGGAAGTGGCGTTGTAACAG	CACTCAGAACGATTAGCATACCTC
2.	CaSnRK2.4	BD	ATGGAAAGATATGAAATTCAGAAAG	TCATAATGCACACACAAAATCACCA
3.	TRV-1		TTACAGGTTATTTGGGCTAG	CCGGGTTCAATTCCTTATC
4.	TRV-2		GGACATTGTTACTCAAGGAAGC	TAAATTACAAAAGACTTACCGATC
5.	T7		TAATACGACTCACTATAGG	
6.	3AD		GAGATGGTGCACGATGCACAGT	
7.	CaSnRK2.4	cLUC	TACGCGTCCCGGGGCGGTACC	TGTAGTCCATTTGTTGGATCC
8.	AtActin		TGTTATGGTAGGGATGGGTC	TTCTCTCTATTTGCCTTGGG
9.	2300		GGGGTACC	GCTCTAGA
10.	BiFC		TGGCGCGCCACTAGTGGATCC	CCGGGAGCGGTACCCTCGAG
11.	cLUC		TACGCGTCCCGGGGCGGTACC	TGTAGTCCATTTGTTGGATCC
12.	nLUC		ACGGGGGACGAGCTCGGTACC	CGCGTACGAGATCTGGTCGAC
13.	CaActin2		TCCACCTCTTCACTCTCTGCTC	TGACCCATCCCTACCATAACAC
14.	35S-GFP		GACGCACAATCCCACTATCC	
15.	CaPDX1 VIGS		ACCATATGATCTGGTGATGCAGACG	CGATTAGCATACCTCTCCACCT

Table S1: Primer’s used during performing experiments**.**
